# Depressive Symptoms, Suicidal Ideation, and Mental Health Service Use of Industrial Workers: Evidence from Vietnam

**DOI:** 10.3390/ijerph17082929

**Published:** 2020-04-23

**Authors:** Ha Ngoc Do, Anh Tuan Nguyen, Hoa Quynh Thi Nguyen, Thanh Phuong Bui, Quy Van Nguyen, Ngan Thu Thi Tran, Long Hoang Nguyen, Hai Quang Pham, Giang Hai Ha, Chi Linh Hoang, Bach Xuan Tran, Carl A. Latkin, Roger C. M. Ho, Cyrus S. H. Ho

**Affiliations:** 1 Youth Research Institute, Ho Chi Minh Communist Youth Union, Hanoi 100000, Vietnam; ngochayri@gmail.com; 2 Department of Research on Youth Culture and Lifestyle, Youth Research Institute, Ho Chi Minh Communist Youth Union, Hanoi 100000, Vietnam; tuananhtwd@gmail.com (A.T.N.); hoaquynh1801@gmail.com (H.Q.T.N.); quynguyen0810@gmail.com (Q.V.N.); 3 Department of Research on Children’s Issues, Youth Research Institute, Ho Chi Minh Communist Youth Union, Hanoi 100000, Vietnam; thanhxhh26@gmail.com; 4 Department of Research on Youth and Legal issues, Youth Research Institute, Ho Chi Minh Communist Youth Union, Hanoi 100000, Vietnam; tranthungan81@gmail.com; 5 VNU School of Medicine and Pharmacy, Vietnam National University, Hanoi 100000, Vietnam; nhlong.smp@vnu.edu.vn; 6 Institute for Global Health Innovations, Duy Tan University, Da Nang 550000, Vietnam; phamquanghai@duytan.edu.vn; 7 Faculty of Medicine, Duy Tan University, Da Nang 550000, Vietnam; 8 Faculty of Pharmacy, Duy Tan University, Da Nang 550000, Vietnam; 9 Center of Excellence in Behavioral Medicine, Nguyen Tat Thanh University, Ho Chi Minh City 700000, Vietnam; chi.coentt@gmail.com (C.L.H.); pcmrhcm@nus.edu.sg (R.C.M.H.); 10 Institute for Preventive Medicine and Public Health, Hanoi Medical University, Hanoi 100000, Vietnam; bach.ipmph@gmail.com; 11 Bloomberg School of Public Health, Johns Hopkins University, Baltimore, MD 21205, USA; carl.latkin@jhu.edu; 12 Department of Psychological Medicine, Yong Loo Lin School of Medicine, National University of Singapore, Singapore 119228, Singapore; 13 Institute for Health Innovation and Technology (iHealthtech), National University of Singapore, Singapore 119077, Singapore; 14 Department of Psychological Medicine, National University Hospital, Singapore 119074, Singapore; cyrushosh@gmail.com

**Keywords:** depressive symptoms, suicidal ideation, industry, worker, Vietnam

## Abstract

**Background**: Depressive symptoms and suicidal ideation substantially reduce industrial workers’ productivity and performance. This study was performed to examine the prevalence of depressive symptoms and suicidal ideation and identify associated factors among industrial workers in different provinces of Vietnam. **Materials and Methods**: We performed a cross-sectional study in industrial zones of four provinces of Vietnam. The Patient Health Questionnaire (PHQ-9) was employed to screen depressive symptoms and suicidal ideation. Multivariate logistic regression was performed to determine factors related to depressive symptoms and suicidal ideation. **Results**: Of 1200 industrial workers, 30.5% and 33.6% industrial workers had positive depressive symptoms and suicidal ideation in the last two weeks, respectively. There were 38.3% ever using mental health services in the last 12 months. High school education (OR = 0.64, 95% CI = 0.44–0.95); living in dormitory (OR = 3.07, 95% CI = 1.51–6.24), living with siblings (OR = 2.98; 95% CI = 1.32–6.75), having two children or more (OR = 1.45, 95% CI = 1.03–2.03), high years of experience (OR = 0.94; 95% CI = 0.89–0.98), suffering from burnout, alcohol use disorder (OR = 2.38; 95% CI = 1.72–3.28), and smoking status (OR = 0.38, 95% CI = 0.23–0.61) were associated with positive depressive symptoms. Living with children, working in mechanical/metallurgy/electronics factories, completely exhausted and often thinking of quitting, and alcohol use disorder were positively related to suicidal ideation. **Conclusions**: This study found a high prevalence of depressive symptoms and suicidal ideation among industrial workers in Vietnam. Regular screening and detecting high-risk groups, along with interventions to reduce health risk behaviors, burnout and on-site medical service quality improvement, are recommended to alleviate the burden of depression in industrial workers.

## 1. Introduction

Depression has been recognized as a great health issue given a dramatic contribution to the disease burden worldwide [1,2]. In the workplace, depression is more common than other mental disorders, and a major cause of reduced productivity, elevated absenteeism and presenteeism, and diminished job retention [3]. Moreover, depressive disorders increase the risk of accidents at work, which results in impaired health conditions, reduced quality of life, suicidal ideation, as well as an additional cost to healthcare and other related problems [3,4,5,6]. It is estimated that a total of depression-attributable costs in the United States is US$36.6 billion per year [7]. This cost is even expected to be significantly higher in developing countries such as China, Bangladesh, and Vietnam, where the economies greatly rely on labor-intensive industries such as textile/shoe-making, electronics, or food processing [8,9].

Industrial workers are among the high-risk populations to depression due to the characteristics of their working process such as a lack of career pathways, hazardous environment exposure, job insecurity, insufficient autonomy, as well as highly monotonous and repetitive tasks [10,11,12,13]. The prevalence of self-reported depression in this population varies regarding nations and industries. For example, the depression rate among garment workers in Bangladesh was 20.9% [8], 31.7% among shoe-making workers in China [14], and 35.4% among automotive assembly workers in Malaysia [11]. In addition, industrial workers were also among the most vulnerable professionals to suicide and suicidal ideation. Studies in the United States, England, and Australia indicated that manual labors (e.g., in the mining or construction industry) had the highest risk of suicide and suicidal ideation compared to other professionals [15,16,17,18]. Another study in Korea found that unskilled male workers had a three times higher rate of suicide-related mortality compared to skilled male workers [19]. Despite these variations, all studies confirmed that the prevalence of depression and suicidal ideation in industrial workers was remarkably higher than that of the general population, as well as the need of the depression screening and risk reduction programs [8,11,14]. These results suggest the necessity of appropriate interventions to screen, control, and improve depression in this vulnerable group.

Both depression and suicidal ideations in industrial workers were associated with multi-level determinants. For example, regarding socio-economic characteristics, higher age, male, higher education level, migrants, and a higher number of health problems were found to be associated with depression in industrial workers [14,20]. Meanwhile, in terms of working conditions, factory workers working in hazardous environments, high work speed, high work-related burnout, as well as having job insecurity, psychological job demand, less decision making participation, and low occupation support had higher levels of depression compared to those without these conditions [11,14,21,22]. Family and social factors were also considered to facilitate depression and suicidal ideation in this population. A study in India found that among female industrial workers, family characteristics such as the number of children, persons living with or support for childcare were significantly associated with suicidal ideation and suicide attempts [23]. Another study in China indicated that poor family relationships were related to depression [14]. However, none of them address the role of the on-site medical facility in improving depression and suicidal ideation in industrial workers [24].

To our knowledge, there is limited evidence about depression and suicidal ideation among industrial workers in Vietnam. A prior study in Hanoi and Bac Ninh indicated that 38.6% of general industrial workers suffered from depressive symptoms [20], while another study showed that 18.8% of workers in shoe-making factories in Hai Phong experienced depression [25]. However, while the former study had a small sample size, the latter focused only on workers in one industry, which might be constrained in interpreting to other industrial populations. Moreover, these studies did not take into account other potential variables such as work-related burnout [22], or the responsiveness of on-site medical services [24]. In order to address these methodological and practical limitations, this study was performed to examine the prevalence of depressive symptoms and suicidal ideation and identify associated factors among industrial workers in different provinces of Vietnam.

## 2. Materials and Methods

### 2.1. Study Design, Sampling Method, and Data Collection

This cross-sectional survey was conducted from January to December 2019 in industrial zones of four provinces including Hanoi, Quang Ngai, Dong Nai, and Can Tho, which were among the places having the largest industrial zones in Vietnam. Factories in these zones manufactured numerous products such as electronics, control devices, shoes, clothes, etc. The sample size for each province was determined by using the formula to estimate a population proportion with specified relative precision:

$$n=Z_{1-\alpha /2}^{2}\frac{1-P}{\varepsilon^{2}P}$$

The parameter for this calculation comprised: a confident level α = 95%, expected prevalence *p* = 38.6% (according to a prior study in Vietnam [26]), relative precision ɛ = 0.15, resulting in 272 workers per province. We added 10% to compensate for people who did not agree to participate or complete the survey for any reason. A total of 300 industrial workers per province, or 1200 samples in total, were invited to become participants of this study (response rate 100%).

Eligible workers were individuals who (1) were aged 18 years or more at the time of the interview; (2) agreed to participate in the study and gave their informed consent. A random sampling method was used to recruit participants. First, we listed all workers in the industrial zones with the support from the managers of the factories. By using computer software, we randomly chose 300 workers in each province. We then contacted them through the introduction of managers and visited their living areas to perform the interview.

Each participant was informed of the study in brief and asked to give their informed consent. They were invited to go to a private room in their accommodation to ensure that their responses were not influenced by other people as well as to protect their privacy. In this study, we used a self-administered paper-based questionnaire to collect data from participants. Industrial workers filled the questionnaire with a pencil assigned from the data collection team. They could also ask the data collector to clarify any questions that they could not understand clearly. Their identifiable information was not collected in the questionnaire in order to facilitate their participation as well as avoid any social desirability bias.

### 2.2. Instruments

The structured questionnaire was designed by the research team, including the following sections: socio-economic status (gender, age, education, marital status, living location, local/migrant people, number of children, and living arrangements), working characteristics (type of factory, years of experience, and working hours per day), depressive symptoms, health status, behaviors, healthcare, and mental health services used.

For screening depressive symptoms, the Patient Health Questionnaire (PHQ-9) was used as a validated tool. Several previous studies were performed that used the Vietnamese version of this instrument to assess the depressive symptoms in different populations [20,26,27]. The PHQ-9 has 9 items about the frequency of depression-related symptoms that the respondents were bothered with in the last two weeks until the interview, which was consistent with the diagnostic standard of the Diagnostic and Statistical Manual of Mental Disorders, 5th Edition (DSM-V). Each item has four responses including “Not at all” (= 0), “Several days” (= 1), “More than half of the days” (= 2), and “Nearly every days” (= 3), resulting in a range score from 0 to 27 [28]. The recommended cut-off points were as follows: normal/minimal (0–4), mild (5–9), moderate (10–14), moderately severe (15–19), and severe (20–27) [28]. Patients having a score of 10 points or more were classified as “positive depressive symptoms” based on its high sensitivity and specificity in previous studies [29,30,31]. Moreover, participants were categorized to have “suicidal ideation” if item 9 “Over the last two weeks, how often have you had thoughts that you would be better off dead or hurting yourself?” had a score of 1 or above. The Cronbach’s alpha was good at 0.889.

Regarding health status, we collected information about whether they had acute symptoms in the last four weeks and chronic diseases in the last three months. Self-rated health was evaluated by using the visual analog scale, with a range score from 0 “The worse health condition that you can imagine” to 100 to “The best health condition that you can imagine”. We also measured the burnout during work of industrial workers by asking them to respond to a single question “How often do you feel exhausted or burnout when working in the factory?” with the answer “Not at all” (=1), “Sometimes being stressed” (= 2), “Definitely exhausted” (= 3), “Exhausted every day and disappointed to my work” (= 4), “Completely exhausted and often thinking of quitting my job” (=5). It is a short, simple, and sensitive screening instrument to evaluate occupational burnout [32].

In terms of behaviors, tobacco and alcohol use practices were evaluated. Participants were asked to report three items of the Alcohol Use Disorders Identification Test-Consumption (AUDIT-C) instrument with a range score from 0 to 12 [33]. Male workers having a score of 4 or more and female workers having a score of 3 or more were categorized as “positive AUDIT-C” [34]. Participants also reported whether they were current smokers or not.

Regarding healthcare and mental health service use, participants were asked to respond to the frequency of regular health examination in the company (none/less than 6 months/6 months/more than 6 months), and the responsiveness of on-site medical services in the company (none/rarely/partly/mostly/completely). Moreover, they were asked to report the use of mental health services in the last 12 months, the facilities and services used.

### 2.3. Statistical Analysis

Statistical significance was detected if a *p*-value was less than 0.05. Descriptive statistical analysis was performed to describe the prevalence of depressive symptoms and suicidal ideation. Chi-squared, Fisher’s exact, and Mann–Whitney tests were then conducted to examine the difference in depressive symptoms and suicidal ideation according to different characteristics. Multivariate logistic regression model was used to identify associations between positive depressive symptoms and suicidal ideation with variables of interests (sociodemographic characteristics, health status, burnout, working characteristics, on-site healthcare service use, and substance use). The model combined with stepwise forward selection strategies to find the optimal results. A log-likelihood test’ *p*-value of less than 0.2 was considered a threshold for selecting variables.

### 2.4. Ethical Approval

The protocol of this study was reviewed and approved by the Institutional Review Board of Youth Research Institute (code: 04d-QD/VNCTN).

## 3. Results

Data of 1200 industrial workers were used for analysis. Table 1 shows the depressive symptoms by socioeconomic and working characteristics. There were 30.5% and 33.6% of industrial workers having positive depressive symptoms and suicidal ideation in the last two weeks, respectively. The rates of normal/minimal, mild, and moderate to severe depressive symptoms were 47.8%, 21.8%, and 30.5%, correspondingly. Significant differences in the rate of depressive symptoms were observed regarding marital status; living location, living with spouse, siblings, or colleagues; years of experience, and working hours per day (*p* < 0.05). Meanwhile, the percentage of industrial workers reporting suicidal ideation was significantly different in education, marital status, local/migrant people, number of children, living with spouse/children/relatives, type of factory, years of experience, and working hours per day (*p* < 0.05).

Table 2 presents that workers reporting positive depressive symptoms or suicidal ideation were more likely to perceived higher levels of burnout during work (*p* < 0.01), and lower self-rated health (*p* < 0.01). The significant differences among depressive severity groups were also found in accordance with having acute symptoms in the last 4 weeks, having chronic diseases in the last 3 months, AUDIT-C positive, and current smoker (*p* < 0.01). Industrial workers having positive depressive symptoms were more likely to perceive less responsiveness of on-site medical services, as well as not receive regular health examination compared to other people (*p* < 0.01). The significant differences of suicidal ideation were also found regarding these characteristics (*p* < 0.01).

Information about mental health service use is presented in Table 3. There were 38.3% ever using mental health services in the last 12 months. Central and provincial hospitals were the most preferred facilities, followed by commune health centers and private hospitals. In addition, mental health counseling and treatment were the two most common services used by the workers.

As presented in Table 4, in the regression models, variables showed independent associations with positive depressive symptoms included high school education (OR = 0.64, 95% CI = 0.44–0.95); living in the dormitory (OR = 3.07, 95% CI = 1.51–6.24), living with siblings (OR = 2.98; 95% CI = 1.32–6.75), having two children or more (OR = 1.45, 95% CI = 1.03–2.03), years of experience (OR = 0.94; 95% CI = 0.89–0.98), burnout during work, AUDIT-C positive (OR = 2.38; 95% CI = 1.72–3.28), and smoking status (OR = 0.38, 95% CI = 0.23–0.61).

In terms of suicidal ideation, higher education, living with a spouse, higher year of experience, having regular examination, and a higher level of responsiveness of on-site medical services were negatively associated with suicidal ideation. Meanwhile, living with children (OR = 2.05, 95% CI = 1.17–3.59), working in mechanical/metallurgy/electronics factories, completely exhausted and often thinking of quitting (OR = 2.80, 95% CI = 1.69–4.65), and AUDIT-C positive were positively related to suicidal ideation.

## 4. Discussion

This study enriches the current literature about a significant burden of depression and suicidal ideation among industrial workers in developing countries such as Vietnam, but mental health care in this population was still lacking. Several factors regarding socio-economic, working, behaviors, and healthcare access were found that increased the vulnerability of industrial workers to depression. These results could suggest further implications to improve the mental health of industrial workers in Vietnam.

In this study, approximately one-third (30.5%) of industrial workers were identified as positive depressive symptoms, which was much higher than that in the Vietnamese general population (2.8%) [35]. Our finding was comparable to a prior finding among industrial workers in Hanoi and Bac Ninh, Vietnam (38.6%—PHQ-9) [20], but approximately 1.5 times higher compared to workers in shoe-making factories in Hai Phong, Vietnam (18.8%) [25]. In addition, this prevalence was relatively higher than that of factory workers in other developing countries such as Bangladesh (20.9% in garment workers) [8] or India (0% in general factory workers but 36% had anxiety disorders and 18% had stress) [35] but equal to the result in China (31.7% in shoe-making workers) [14] and Malaysia (35.4% in automotive assembly workers) [11]. Notably, our result indicated that one-third of our sample had suicidal ideation in the last two weeks, which was more than four times higher when compared with the rate of suicidal ideation in the general Vietnamese population (8.9%) [36]. This prevalence was significantly higher than that in other settings such as the United States (2.6%) [37] and Korea (2.8%) [38]. Prior literature revealed that industrial workers were particularly vulnerable populations to depression and other mental health disorders given their low education and income, highly repetitive work, and constrained social support [6,39]. As mental problems significantly impact workforces’ productivity [3], this finding raises the urgent need for regular mental health screening and interventions to improve the health and performance of industrial workers.

Furthermore, the result indicated that only one-third of individuals having positive depressive symptoms used mental care in the last 12 months. This finding suggested that (1) only a limited number of workers with depression could access appropriate mental health services, or (2) more industrial workers suffered from depression in the last 12 months compared to our findings, and their illness was improved before the survey. Moreover, we observed that provincial and central hospitals were the most preferred health facilities the industrial workers visited for general counseling or treatment. Meanwhile, we did not find any industrial workers who reported using medical services in the factory for mental care. This phenomenon can be explained by the fact that the workers might be afraid of discrimination at work or even to be fired if they disclosed their mental status, which has been shown in prior literature [40,41,42]. Another reason is that mental health-related physicians, infrastructure, or equipment in the on-site medical offices was insufficient to consult, diagnose, or treat depression [34]. Of note, our regression analysis revealed a critical role of on-site medical services on improving the mental health of industrial workers. Specifically, having a regular health examination, as well as high responsiveness degree of the medical office in the factory, were associated with a lower risk of suicidal ideation or marginally related to the lower likelihood of being depressed. These results suggested on-site medical services should be enhanced to ensure meeting the needs for workers in physical and psychological care during their work.

Our study found associations between socio-demographic characteristics with depressive symptoms and suicidal ideation, suggesting vulnerable populations that should be paid attention. For example, people with a higher education were less likely to be depressed or have suicidal ideation, aligning with previous studies that a high level of education was correlated with mental resources preparedness, which increased the individual’s resilience to cope with stress and depression [25,43]. Moreover, our finding revealed that living in a dormitory significantly increased the risk of depressive symptoms. In fact, most of the workers living in this accommodation were migrants from other locals, who might have poor family support or connection [39,44]. On the other hand, living with spouses was associated with a lower likelihood of suicidal ideation, one of the depressive symptoms, confirming the important role of family support in reducing the risk of depressive symptoms as in previous studies [20,25]. However, living with children, especially two children or above, significantly increased the risk of being depressed. This might be justified by the fact that industrial workers in Vietnam had relatively low income because they mostly participated in manual work [45]. Thus, having children could result in a heavy financial burden for the workers, increasing the risk of depression [8].

Furthermore, data from our sample suggested that people working in mechanical/metallurgy, electronics, and food processing factories had a higher likelihood of being depressed compared to those in textile/shoe-making factories. Indeed, workers in the three former factories were mostly assigned to perform the task in the assembly line and complete the product according to a standardized procedure [46]. However, doing this work is very challenging because it is monotonous and repetitive but requires concentration in a long period of time [12]. Thus, workers were frequently overloaded and more likely to suffer workplace stress and depression [10]. We also found a remarkable association between burnout and depressive symptoms. This result was in line with a study among Chinese factory workers, which indicated that in comparison with workers without burnout, workers with mild, moderate, to severe burnout were 1.4, 3.8, and 25.5 times more likely to suffer from psychological disorders [47]. Burnout refers to mental exhaustion at the workplace due to stressful working environments, spending high efforts but poor satisfaction with work [48]. It has been shown that it can negatively affect the psychological health of the individual, leading to the development of depression [48].

Results in our study were congruent with previous studies that smoking was associated with a lower risk of positive depressive symptoms [20,49]. Practically, smoking could be concerned among industrial workers as a coping strategy to reduce the depressive symptoms via its pleasure effects [50,51]. A prior review indicated that depressive symptoms were associated with higher levels of cigarette use, as well as were considered an obstacle for smoking cessation and smoking abstinence [52]. Moreover, we echoed existing literature about the negative effect of alcohol abuse, since positive AUDIT-C was associated with depressive symptoms as well as suicidal ideation among industrial workers. Due to the nature of cross-sectional design, we could not confirm that alcohol abuse resulted in depression or depression led to alcohol abuse; however, a previous review suggested that alcohol use disorders were more likely to be a risk factor for the onset of depression rather than vice versa [53,54]. Therefore, pharmacological and behavioral interventions should be developed and performed to diminish the risk of smoking and alcohol use as well as improve the mental health of industrial workers.

## 5. Conclusions

To conclude, this study found a high prevalence of depressive symptoms and suicidal ideation among industrial workers in Vietnam. Moreover, we found that substance use, types of industry, work-related burnout, regular health examination, and high responsiveness of on-site medical service were significantly correlated with depressive symptoms and suicidal ideations among industrial workers in Vietnam.

The findings of this study suggested several implications. Given the high burden of depressive symptoms in industrial workers, regular screening and detecting high-risk populations such as people with low education, living with children, substance use, and experiencing burnout would be critical to offering timely counseling and treatment. Some work (years of experience or burnout) and behavior (alcohol use or smoking) -related factors could serve as indicators to guide the promotion interventions in the workplace. On-site medical services should be improved to meet the need for mental care among industrial workers. Finally, changing positions and duties appropriately and providing positive feedback for people’s work should be concerned to reduce the workers’ burnout.

Our strengths included a large sample size in different provinces in Vietnam. Compared to other previous studies in Vietnam (with 289 [20] and 420 workers [25]), we recruited 1200 industrial workers in four provinces in all three main regions of Vietnam: northern (Hanoi), middle (Quang Ngai), and southern (Dong Nai and Can Tho) regions. Moreover, we employed international validated instruments such as PHQ9 and AUDIT-C, which improved our comparability to other studies in the world. The limitations of this study include the use of a cross-sectional design which hindered our ability to conclude causation. Moreover, although PHQ-9 is a validated instrument for screening depression, it cannot be used for diagnosis. Additionally, the PHQ-9 only investigated depressive symptoms and suicidal ideation in the last two weeks; thus, it is believed that the 12-month prevalence of these problems might be much higher than we observed in this study [28,31]. Our data was mainly based on self-reported information, which might result in social desirability bias. Regarding the burnout measure, although our approach in using only a single question might have a lower validity compared to other well-structured instruments for this topic, it was suggested to use for screening at-risk populations for suicide [55].

## Figures and Tables

**Table 1:** Depressive symptoms by socioeconomic and working characteristics.

Characteristics	Total	Positive Depressive Symptoms		Suicidal Ideation	
	n	n (%)	*p*-Value	n (%)	*p*-Value
Total (n = 1200)	1200	366 (30.5)		403 (33.6)	
Gender (n = 1200)					
Male	530	155 (29.3)	0.40	191 (36.0)	0.11
Female	670	211 (31.5)		212 (31.6)	
Education (n = 1200)					
<High school	217	66 (30.4)	0.03	86 (39.6)	<0.01
High school	509	135 (26.5)		134 (26.3)	
Vocational training/college	237	77 (32.5)		104 (43.9)	
University	237	88 (37.1)		79 (33.3)	
Marital status (n = 1200)					
Single	530	144 (27.2)	0.03	139 (26.2)	<0.01
Married	670	222 (33.1)		264 (39.4)	
Living location (n = 1200)					
House	782	231 (29.5)	<0.01	252 (32.2)	0.36
Homestay	376	113 (30.1)		137 (36.4)	
Dormitory	42	22 (52.4)		14 (33.3)	
Local people (n = 1200)					
Yes	904	267 (29.5)	0.21	284 (31.4)	<0.01
No	296	99 (33.5)		119 (40.2)	
Number of children (n = 1200)					
0	611	168 (27.5)	0.06	192 (31.4)	0.04
1	301	98 (32.6)		119 (39.5)	
≥2	288	100 (34.7)		92 (31.9)	
Living arrangements (n = 1200)					
Parents *	546	164 (30.0)	0.75	175 (32.1)	0.31
Spouse *	586	198 (33.8)	0.02	233 (39.8)	<0.01
Children *	265	82 (30.9)	0.86	38 (14.3)	<0.01
Siblings *	53	9 (17.0)	0.03	23 (43.4)	0.12
Relatives *	44	14 (31.8)	0.85	7 (15.9)	0.01
Colleagues *	74	15 (20.3)	0.04	19 (25.7)	0.14
Other *	37	11 (29.7)	0.92	7 (18.9)	0.06
Type of industry (n = 1200)					
Textile/Shoe-making	582	156 (26.8)	0.03	74 (12.7)	<0.01
Mechanical/metallurgy	181	61 (33.7)		123 (68.0)	
Electronics	200	74 (37.0)		148 (74.0)	
Food processing	146	41 (28.1)		54 (37.0)	
Other	91	34 (37.4)		4 (4.4)	
Years of experience (n = 1200)					
≤1 year	141	38 (27.0)	<0.01	66 (46.8)	<0.01
2–5 years	840	280 (33.3)		327 (38.9)	
>5 years	219	48 (21.9)		10 (4.6)	
Working hours per day (n = 1200)					
8 h	1001	305 (30.5)	0.96	315 (31.5)	<0.01
>8 h	199	61 (30.7)		88 (44.2)	

* Comparisons between those living and those not living with each type of person.

**Table 2:** Depressive symptoms according to health status, behaviors, and on-site health care.

Characteristics	Total	Positive Depressive Symptoms		Suicidal Ideation	
	n	n (%)	*p*-Value	n (%)	*p*-Value
Burnout during work (n = 1200)					
Not at all	348	45 (12.9)	<0.01	77 (22.1)	<0.01
Sometimes being stressed	367	120 (32.7)		33 (9.0)	
Definitely exhausted	25	15 (60.0)		7 (28.0)	
Exhausted everyday	200	98 (49.0)		87 (43.5)	
Completely exhausted and often thinking of quitting	260	88 (33.9)		199 (76.5)	
Having acute symptoms in the last 4 weeks (n = 1200)					
No	561	159 (28.3)	0.13	184 (32.8)	0.59
Yes	639	207 (32.4)		219 (34.3)	
Having chronic diseases in the last 3 months (n = 1200)					
No	535	148 (27.7)	0.06	164 (30.7)	0.054
Yes	665	218 (32.8)		239 (35.9)	
AUDIT-C positive (n = 1200)					
No	784	214 (27.3)	<0.01	236 (30.1)	<0.01
Yes	416	152 (36.5)		167 (40.1)	
Current smoker (n = 1200)					
No	1034	334 (32.3)	<0.01	351 (34.0)	0.51
Yes	166	32 (19.3)		52 (31.3)	
Frequency of regular health examination (n = 1200)					
None	90	26 (28.9)	<0.01	70 (77.8)	<0.01
<6 months	147	50 (34.0)		55 (37.4)	
6 months	511	102 (20.0)		140 (27.4)	
>6 months	452	188 (41.6)		138 (30.5)	
Responsiveness of on-site medical services (n = 1200)					
None	5	1 (20.0)	<0.01	0 (0.0)	<0.01
Rarely	27	11 (40.7)		7 (25.9)	
Partly	178	45 (25.3)		59 (33.2)	
Mostly	549	200 (36.4)		254 (46.3)	
Completely	441	109 (24.7)		83 (18.8)	
	n	Mean (SD)	*p*-value	Mean (SD)	*p*-value
Visual Analogue Scale (0–100) *	1200	47.4 (20.9)		49.4 (21.4)	<0.01

* Comparison between those with and without positive depressive symptoms/suicidal ideation.

**Table 3:** Healthcare and mental health service use.

Characteristics	Total	Depressive Severity			
		Normal/Minimal	Mild	Moderate to Severe	*p*-Value
	n	n (%)	n (%)	n (%)	
Using mental health service in the last 12 months (n = 1200)					
No	741	349 (60.9)	160 (61.3)	232 (63.4)	0.74
Yes	459	224 (39.1)	101 (38.7)	134 (36.6)	
Mental health facility used (n = 459)					
Commune health center	75	32 (14.3)	15 (14.9)	28 (20.9)	0.24
District hospitals	24	10 (4.5)	4 (4.0)	10 (7.5)	0.38
Provincial hospitals	328	163 (72.8)	72 (71.3)	93 (69.4)	0.79
Central hospitals	342	176 (78.6)	78 (77.2)	88 (65.7)	0.02
Private hospitals	33	13 (5.8)	8 (7.9)	12 (9.0)	0.51
Others	10	4 (1.8)	3 (3.0)	3 (2.2)	0.79
Mental health services used (n = 459)					
General mental health counseling	319	156 (69.6)	74 (73.3)	89 (66.4)	0.53
Occupational stress	57	31 (13.8)	7 (6.9)	19 (14.2)	0.17
Mental health treatment	166	72 (32.1)	31 (30.7)	63 (47.0)	<0.01
Others	20	9 (4.0)	2 (2.0)	9 (6.7)	0.20

**Table 4:** Associated factors with depressive symptoms and suicidal ideation.

Characteristics	Positive Depressive Symptoms		Suicidal Ideation	
	OR ^1^	95% CI ^2^	OR	95% CI
Individual characteristics				
Education				
<High school	ref ^3^	-	ref	-
High school	0.64 **	0.44; 0.95	0.38 ***	0.23; 0.62
Vocational training/college	0.89	0.58; 1.37	0.55 **	0.32; 0.96
University	1.10	0.72; 1.70	0.69	0.39; 1.22
Visual analogue scale	0.99 *	0.99; 1.00	0.98 ***	0.97; 0.99
AUDIT-C positive				
No	ref	-	ref	-
Yes	2.38 ***	1.72; 3.28	1.82 ***	1.26; 2.63
Current smoker				
No	ref	-		
Yes	0.38 ***	0.23; 0.61		
Family and social characteristics				
Living location				
House	ref	-		
Homestay	1.00	0.74; 1.36		
Dormitory	3.07 ***	1.51; 6.24		
Living arrangements (Yes vs. No-ref)				
Spouse			0.50 ***	0.34; 0.73
Children			2.05 **	1.17; 3.59
Siblings	2.98 ***	1.32; 6.75		
Relatives			2.64 *	0.93; 7.50
Number of children				
0	ref	-		
1	1.19	0.86; 1.66		
≥2	1.45 **	1.03; 2.03		
Local people				
Yes			ref	-
No			1.36	0.92; 2.01
Working characteristics				
Type of industry				
Textile/Shoe-making			ref	-
Mechanical/metallurgy			4.65 ***	2.75; 7.87
Electronics			5.11 ***	3.00; 8.70
Food processing			1.80 **	1.02; 3.18
Other			0.26 **	0.09; 0.81
Years of experience	0.94 ***	0.89; 0.98	0.79 ***	0.71; 0.87
Working hours per day			1.29 *	0.97; 1.72
Burnout during work				
Not at all	ref	-	ref	-
Sometimes being stressed	2.76 ***	1.80; 4.23	0.80	0.47; 1.37
Definitely exhausted	11.81 ***	4.73; 29.51	1.85	0.65; 5.28
Exhausted everyday	3.59 ***	2.28; 5.67	1.11	0.66; 1.86
Completely exhausted and often thinking of quitting	1.82 **	1.15; 2.88	2.80 ***	1.69; 4.65
Healthcare use characteristics				
Frequency of regular health examination (n = 1200)				
None	ref	-	ref	-
<6 months	1.52	0.81; 2.85	0.65	0.30; 1.41
6 months	0.62	0.35; 1.10	0.33 ***	0.17; 0.67
>6 months	1.59 *	0.92; 2.76	0.29 ***	0.14; 0.57
Responsiveness degree of on-site medical services	0.84 *	0.71; 1.01	0.74 ***	0.59; 0.92

*** *p* < 0.01; ** *p* < 0.05; * *p* < 0.1; ^1^ odds ratio; ^2^ confidence interval; ^3^ reference group.

## References

[1] Murray C.J.L., Vos T., Lozano R., Naghavi M., Flaxman A.D., Michaud C., Ezzati M., Shibuya K., Salomon J.A., Abdalla S. (2012). Disability-adjusted life years (DALYs) for 291 diseases and injuries in 21 regions, 1990–2010: A systematic analysis for the Global Burden of Disease Study 2010. Lancet.

[2] Chisholm D., Sanderson K., Ayuso-Mateos J.L., Saxena S. (2004). Reducing the global burden of depression: Population-level analysis of intervention cost-effectiveness in 14 world regions. Br. J. Psychiatry J. Ment. Sci..

[3] Burton Wayne N. (2008). The association of worker productivity and mental health: A review of the literature. Int. J. Workplace Health Manag..

[4] Rajgopal T. (2010). Mental well-being at the workplace. Indian J. Occup. Environ. Med..

[5] World Health Organization (2004). Work Organizing and Stress Protecting Workers’.

[6] Lau J.T.F., Cheng Y., Gu J., Zhou R., Yu C., Holroyd E., Yeung N.C.Y. (2012). Suicides in a mega-size factory in China: Poor mental health among young migrant workers in China. Occup. Environ. Med..

[7] Lépine J.-P., Briley M. (2011). The increasing burden of depression. Neuropsychiatr. Dis. Treat..

[8] Fitch T.J., Moran J., Villanueva G., Sagiraju H.K.R., Quadir M.M., Alamgir H. (2017). Prevalence and risk factors of depression among garment workers in Bangladesh. Int. J. Soc. Psychiatry.

[9] United Nations Industrial Development Organization (2015). Industrial Parks, Special Economics Zones, eco Industrial Parks, Innovation Districts as Strategies for Industrial Conpetitiveness. Economic Zones in the Asean.

[10] Lundberg U., Granqvist M., Hansson T., Magnusson M., Wallin L. (1989). Psychological and physiological stress responses during repetitive work at an assembly line. Work Stress.

[11] Edimansyah B.A., Rusli B.N., Naing L., Mohamed Rusli B.A., Winn T., Tengku Mohamed Ariff B.R.H. (2008). Self-perceived depression, anxiety, stress and their relationships with psychosocial job factors in male automotive assembly workers. Ind. Health.

[12] Ji Y., Li S., Wang C., Wang J., Liu X. (2016). Occupational stress in assembly line workers in electronics manufacturing service and related influencing factors. Chin. J. Ind. Hyg. Occup. Dis..

[13] Dianat I., Salimi A. (2014). Working conditions of Iranian hand-sewn shoe workers and associations with musculoskeletal symptoms. Ergonomics.

[14] Ren F., Yu X., Dang W., Niu W., Zhou T., Lin Y., Wu Z., Lin L., Zhong B., Chu H. (2019). Depressive symptoms in Chinese assembly-line migrant workers: A case study in the shoe-making industry. Asia Pac. Psychiatry.

[15] Kposowa A.J. (1999). Suicide mortality in the United States: Differentials by industrial and occupational groups. Am. J. Ind. Med..

[16] Windsor-Shellard B., Gunnell D. (2019). Occupation-specific suicide risk in England: 2011–2015. Br. J. Psychiatry.

[17] Heller T.S., Hawgood J.L., Leo D.D. (2007). Correlates of suicide in building industry workers. Arch. Suicide Res. Off. J. Int. Acad. Suicide Res..

[18] Roberts S.E., Jaremin B., Lloyd K. (2013). High-risk occupations for suicide. Psychol. Med..

[19] Yoon J.H., Jung S.J., Choi J., Kang M.Y. (2019). Suicide Trends over Time by Occupation in Korea and Their Relationship to Economic Downturns. Int. J. Environ. Res. Public Health.

[20] Tran B.X., Vu G.T., Pham K.T.H., Vuong Q.H., Ho M.T., Vuong T.T., Nguyen H.T., Nguyen C.T., Latkin C.A., Ho C.S.H. (2019). Depressive Symptoms among Industrial Workers in Vietnam and Correlated Factors: A Multi-Site Survey. Int. J. Environ. Res. Public Health.

[21] Boschman J.S., van der Molen H.F., Sluiter J.K., Frings-Dresen M.H. (2013). Psychosocial work environment and mental health among construction workers. Appl. Ergon..

[22] Yeh S.S., Lee C.N., Wu Y.H., Tu N.C., Guo Y.L., Chen P.C., Chen C.H. (2018). Occupational Hazard Exposures and Depressive Symptoms of Pregnant Workers. J. Occup. Environ. Med..

[23] Travasso S.M., Rajaraman D., Heymann S.J. (2014). A qualitative study of factors affecting mental health amongst low-income working mothers in Bangalore, India. BMC Women Health.

[24] Yates D.W., Peters K. (1983). Do on-site medical facilities influence the outcome of industrial accidents?. J. Soc. Occup. Med..

[25] Minh K.P. (2014). Work-related depression and associated factors in a shoe manufacturing factory in Haiphong City, Vietnam. Int. J. Occup. Med. Environ. Health.

[26] Pham T., Bui L., Nguyen A., Nguyen B., Tran P., Vu P., Dang L. (2019). The prevalence of depression and associated risk factors among medical students: An untold story in Vietnam. PLoS ONE.

[27] Nguyen L.H., Tran B.X., Nguyen N.P., Phan H.T., Bui T.T., Latkin C.A. (2016). Mobilization for HIV Voluntary Counseling and Testing Services in Vietnam: Clients’ Risk Behaviors, Attitudes and Willingness to Pay. AIDS Behav..

[28] Spitzer R., Williams J., Kroenke K.J.R.C.B. (2014). Test Review: Patient Health Questionnaire–9 (PHQ-9). Rehabil. Couns. Bull..

[29] Manea L., Gilbody S., McMillan D. (2015). A diagnostic meta-analysis of the Patient Health Questionnaire-9 (PHQ-9) algorithm scoring method as a screen for depression. Gen. Hosp. Psychiatry.

[30] Gilbody S., Richards D., Brealey S., Hewitt C. (2007). Screening for depression in medical settings with the Patient Health Questionnaire (PHQ): A diagnostic meta-analysis. J. Gen. Intern. Med..

[31] Kroenke K., Spitzer R.L., Williams J.B. (2001). The PHQ-9: Validity of a brief depression severity measure. J. Gen. Intern. Med..

[32] Hansen V., Pit S. (2016). The Single Item Burnout Measure is a Psychometrically Sound Screening Tool for Occupational Burnout. Health Scope.

[33] Bradley K.A., DeBenedetti A.F., Volk R.J., Williams E.C., Frank D., Kivlahan D.R. (2007). AUDIT-C as a brief screen for alcohol misuse in primary care. Alcohol. Clin. Exp. Res..

[34] Vuong D.A., Van Ginneken E., Morris J., Ha S.T., Busse R. (2011). Mental health in Vietnam: Burden of disease and availability of services. Asian J. Psychiatry.

[35] Rao S., Ramesh N. (2015). Depression, anxiety and stress levels in industrial workers: A pilot study in Bangalore, India. Ind. Psychiatry J..

[36] Tran Thi Thanh H., Tran T.N., Jiang G.-X., Leenaars A., Wasserman D. (2006). Life time suicidal thoughts in an urban community in Hanoi, Vietnam. BMC Public Health.

[37] Han B., Crosby A.E., Ortega L.A., Parks S.E., Compton W.M., Gfroerer J. (2016). Suicidal ideation, suicide attempt, and occupations among employed adults aged 18-64years in the United States. Compr. Psychiatry.

[38] Kang M.-Y., Kwon H.-J., Choi K.-H., Kang C.-W., Kim H. (2017). The relationship between shift work and mental health among electronics workers in South Korea: A cross-sectional study. PLoS ONE.

[39] Zhong B.-L., Liu T.-B., Chan S.S.M., Jin D., Hu C.-Y., Dai J., Chiu H.F.K. (2015). Prevalence and correlates of major depressive disorder among rural-to-urban migrant workers in Shenzhen, China. J. Affect. Disord..

[40] Maulik P.K. (2017). Workplace stress: A neglected aspect of mental health wellbeing. Indian J. Med Res..

[41] Brouwers E.P.M., Mathijssen J., Van Bortel T., Knifton L., Wahlbeck K., Van Audenhove C., Kadri N., Chang C., Goud B.R., Ballester D. (2016). Discrimination in the workplace, reported by people with major depressive disorder: A cross-sectional study in 35 countries. BMJ Open.

[42] Brouwers E.P.M., Joosen M.C.W., van Zelst C., Van Weeghel J. (2019). To Disclose or Not to Disclose: A Multi-stakeholder Focus Group Study on Mental Health Issues in the Work Environment. J. Occup. Rehabil..

[43] Bjelland I., Krokstad S., Mykletun A., Dahl A.A., Tell G.S., Tambs K. (2008). Does a higher educational level protect against anxiety and depression? The HUNT study. Soc. Sci. Med..

[44] Silveira E.R., Ebrahim S. (1998). Social determinants of psychiatric morbidity and well-being in immigrant elders and whites in east London. Int. J. Geriatr. Psychiatry.

[45] Research S. (2019). Vietnam Industrial H1/2019.

[46] Yu S.-F., Jiang K.-Y., Zhou W.-H., Sheng W.J.C.M.J. (2008). Relationship between occupational stress and salivary sIgA and lysozyme in assembly line workers. Chin. Med. J..

[47] Lu Y., Zhang Z., Gao S., Yan H., Zhang L., Liu J. (2020). The Status of Occupational Burnout and Its Influence on the Psychological Health of Factory Workers and Miners in Wulumuqi, China. BioMed Res. Int..

[48] Iacovides A., Fountoulakis K.N., Kaprinis S., Kaprinis G. (2003). The relationship between job stress, burnout and clinical depression. J. Affect. Disord..

[49] Rosario M., Schrimshaw E.W., Hunter J. (2011). Cigarette smoking as a coping strategy: Negative implications for subsequent psychological distress among lesbian, gay, and bisexual youths. J. Pediatric Psychol..

[50] Weinberger A.H., George T.P., McKee S.A. (2011). Differences in smoking expectancies in smokers with and without a history of major depression. Addict. Behav..

[51] Currie S.R., Hodgins D.C., el-Guebaly N., Campbell W. (2001). Influence of depression and gender on smoking expectancies and temptations in alcoholics in early recovery. J. Subst. Abus..

[52] Weinberger A.H., Kashan R.S., Shpigel D.M., Esan H., Taha F., Lee C.J., Funk A.P., Goodwin R.D. (2017). Depression and cigarette smoking behavior: A critical review of population-based studies. Am. J. Drug Alcohol Abus..

[53] Boden J.M., Fergusson D.M. (2011). Alcohol and depression. Addiction.

[54] Schuckit M.A. (1994). Alcohol and depression: A clinical perspective. Acta Psychiatr. Scand. Suppl..

[55] The Joint Commission (2016). Sentinel Event Alert 56: Detecting and Treating sUicide Ideation in All Settings.

